# Validation of the IHE Cohort Model of Type 2 Diabetes and the Impact of Choice of Macrovascular Risk Equations

**DOI:** 10.1371/journal.pone.0110235

**Published:** 2014-10-13

**Authors:** Adam Lundqvist, Katarina Steen Carlsson, Pierre Johansen, Emelie Andersson, Michael Willis

**Affiliations:** 1 The Swedish Institute for Health Economics, IHE, Lund, Sweden; 2 Department of Clinical Sciences, Lund University, Malmö, Sweden; Old Dominion University, United States of America

## Abstract

**Background:**

Health-economic models of diabetes are complex since the disease is chronic, progressive and there are many diabetic complications. External validation of these models helps building trust and satisfies demands from decision makers. We evaluated the external validity of the IHE Cohort Model of Type 2 Diabetes; the impact of using alternative macrovascular risk equations; and compared the results to those from microsimulation models.

**Methods:**

The external validity of the model was analysed from 12 clinical trials and observational studies by comparing 167 predicted microvascular, macrovascular and mortality outcomes to the observed study outcomes. Concordance was examined using visual inspection of scatterplots and regression-based analysis, where an intercept of 0 and a slope of 1 indicate perfect concordance. Additional subgroup analyses were conducted on ‘dependent’ vs. ‘independent’ endpoints and microvascular vs. macrovascular vs. mortality endpoints.

**Results:**

Visual inspection indicates that the model predicts outcomes well. The UKPDS-OM1 equations showed almost perfect concordance with observed values (slope 0.996), whereas Swedish NDR (0.952) and UKPDS-OM2 (0.899) had a slight tendency to underestimate. The *R* ^2^ values were uniformly high (>0.96). There were no major differences between ‘dependent’ and ‘independent’ outcomes, nor for microvascular and mortality outcomes. Macrovascular outcomes tended to be underestimated, most so for UKPDS-OM2 and least so for NDR risk equations.

**Conclusions:**

External validation indicates that the IHE Cohort Model of Type 2 Diabetes has predictive accuracy in line with microsimulation models, indicating that the trade-off in accuracy using cohort simulation might not be that large. While the choice of risk equations was seen to matter, each were associated with generally reasonable results, indicating that the choice must reflect the specifics of the application. The largest variation was observed for macrovascular outcomes. There, NDR performed best for relatively recent and well-treated patients, while UKPDS-OM1 performed best for the older UKPDS cohort.

## Introduction

Economic evaluation identify the likely health and cost consequences of proposed treatment interventions and is necessary for making health care decisions that allocate limited resources efficiently [1]. Economic modelling is used and widely accepted in economic evaluation, especially for chronic and progressive diseases like Type 2 Diabetes Mellitus (T2DM), where long time horizons are required to realize the full costs and consequences of intervention and where registration-oriented clinical trials are often relatively short [2], [3]. Economic simulation models are constructed with sets of mathematical equations that synthesize the available data (frequently from multiple sources) into a coherent and internally consistent framework. Data sources include short-run clinical trial for treatment effects and adverse event rates, risk equations and known physiological relationships to project patient outcomes over time, and unit cost and quality of life weights from registry data and surveys.

From a modelling standpoint, T2DM ranks among the most challenging of disease areas, as it affects multiple inter-related organ systems (e.g., cardiovascular disease, nephropathy, neuropathy, and nephropathy); these complications often take years to develop and the event rates tend to accelerate over time; and co-morbid conditions such as hypertension, dyslipidemia, and obesity are common [2]. Moreover, treatment is routinely multi-factorial, treatments for different co-morbid conditions frequently work on the same set of risk factors, and treatments often have limited durability and treatment intensification over time is routine.

Given the level of detail in models of T2DM intervention, the users of model results need to be convinced of the soundness of model-based predictions. To help allay concerns, the International Society for Pharmacoeconomics and Outcomes Research (ISPOR) and the Society for Medical Decision Making (SMDM) have jointly issued good practice recommendations for model validation [4]. They emphasize the assurance of *face validity* (i.e., that the model reflects current scientific evidence as judged by experts), *verification* (i.e., de-bugging, ‘stress-testing’, and other activities that ensure model calculations are correctly implemented), *cross-validation* (i.e., assessing whether different models generate similar results to a standardized study question), and *external* (and *predictive*) *validation* (i.e., testing whether the model accurately predicts actual outcomes observed in patients in clinical trials or observational registries) [5].

The IHE Cohort Model of Type 2 Diabetes is a new deterministic, cohort-level cost-effectiveness model of treatment intervention in T2DM. Briefly, it is constructed of Markov health states that comprise the key diabetic complications associated with T2DM: microvascular complications (retinopathy, nephropathy, and neuropathy) and macrovascular complications (myocardial infarction (MI), ischemic heart disease (IHD), congestive heart failure (CHF), stroke, and peripheral vascular disease (PVD)). Progression to more severe health states is evaluated on annual basis based on event risks that are tailored to the current values of time-varying cohort characteristics (e.g., age, gender, disease duration, and key bio-markers such as HbA1c and systolic blood pressure) using risk equations. For macrovascular disease, the user can choose between the original United Kingdom Prospective Diabetes Study Outcomes Model (UKPDS-OM1) [6], the revised UKPDS Outcomes Model (UKPDS-OM2) [7], or the Swedish National Diabetes Register (NDR) risk equations [8]. Microvascular complication risks are largely as in the seminal National Institutes of Health (NIH) model [9] as updated in the DiDACT model [10]. Treatment interventions are applied initially and updated over the course of the user-specified time horizon (maximum of 40 years) to meet pre-specified HbA1c goals, generating treatment-arm-specific health profiles and hence differences in micro- and macrovascular outcomes. Unit costs and QALY disutility weights are applied to the cohort outcomes and summed, then the cost-effectiveness ratio and net monetary benefits are calculated. Uncertainty in the model parameters, often called 2^nd^ order uncertainty, is captured with the inclusion of (optional) probabilistic sensitivity analysis (PSA). Further description of the model is available in Supporting Information: File S1.

Most models of T2DM have adopted the micro-simulation (i.e., patient-level) approach, including, for example, 10 of the 12 models adopted by Tarride and colleagues [11] and 7 of the 8 models participating at an international congress for economic models of T2DM (the 5^th^ Mt. Hood Challenge) [12]. As noted in the ISPOR/T2DM modeling recommendations, micro-simulation models are better able to track complex disease histories and thus account for interdependence in complications (which is important in multi-organ-system diseases such as T2DM) [13]. The downsides of micro-simulation models in T2DM, however, relate to the complexity required and the often long run times required to generate robust simulation results. The IHE Cohort Model of Type 2 Diabetes uses the cohort approach because it was easier to develop (reducing the risk of programming or logical errors), debug, and communicate to non-experts. Moreover, the run times for The IHE Cohort Model of Type 2 Diabetes are short when compared to most micro-simulation models of T2DM (which frequently run in hours), which is an advantage in evaluating T2DM interventions where hundreds of simulations are routinely required (given multiple indications and treatment comparators and the need for extensive sensitivity analysis).

It is important to ensure that these potential benefits are not associated with a reduction in performance versus micro-simulation models. The objective of this paper, thus, is to test the external validity (i.e., accuracy of model predictions versus actual observed outcomes) of the IHE Cohort Model of Type 2 Diabetes. In addition, we use the opportunity to explore differences in predictive accuracy for the three sets of competing macrovascular risk equations.

## Materials and Methods

We test the external validation of The IHE Cohort Model of Type 2 Diabetes according to the recommendations of ISPOR/MSDM [5]. Because the model is claimed as a general multi-application ‘general diabetes’ model, it was not calibrated to any of the individual studies included in the validation exercises and the same model version was used for each validation exercise. Specifically, our methodology consisted of:

- Identify a suitable sample of validation studies to replicate. Studies were selected to provide a distribution geographically, trial vs. non-interventional observational naturalistic data collection, studies used in model construction (“dependent”) vs. not used in model construction (“independent”), and studies used in other validation examples in T2DM.
- Load the IHE Cohort Model of Type 2 Diabetes the with mean baseline patient (demographic and clinical) characteristics of each of the included validation studies (including sub-groups where applicable), one study at a time. In some instances, the published material did not include information on specific model parameters and we used corresponding data from studies with similar patient populations (Supporting Information: Table S1). The model was loaded separately for 4 sub-groups with important CVD risk implications (female smokers, female non-smokers, male smokers, and male non-smokers) and the weighted average was calculated for each outcome.
- Load the model with the effects of intervention (if any) and the changes in bio-marker parameters over time. We assumed that treatment effects reported from trials reached full effect during the first year.
- Simulate the scenario for the same time horizon as the mean duration of follow-up in the study for each of the four sub-groups described above, extract each of the outcomes (primary and secondary) in the study that could be matched with output from the IHE Cohort Model of Type 2 Diabetes, and calculate the weighted averages.
- To examine potential differences related to choice of macrovascular risk equations, we simulated each of the validation studies three times using the UKPDS-OM1, UKPDS-OM2, and Swedish NDR sets of risk equations (but with everything else the same).
- Concordance between model predictions and the actual observed cumulative incidence outcomes was evaluated graphically as a scatterplot and by estimating the best-fitting linear regression line, both for the full sample and for important sub-groups of outcomes (see details below).

### Validation Studies

In line with previous validation studies [14]–[18] and the Mt. Hood Challenges [12], [19], 12 studies were selected for the analysis, including both clinical trials and observational naturalistic studies. Collectively, the selected studies enrolled nearly 90,000 subjects. The studies are summarized in Table 1.

**Table 1:** Studies included in the validation analyses.

Study name	Population	Treatmentgroups	Duration(years)	Participants	Endpoints
NDR (I) [8]	Observational study of residentsin Sweden diagnosed with type2 diabetes at an age of 30–75,followed from 2003	Observational	5	29,034	32
NDR (II) [22]	Observational study of residentsin Sweden with type 2 diabetes,ages 30–79, followed from 1997/1998	Observational	5.6	18,334	3
UKPDS 33 [23]	Interventional study of newlydiagnosed type 2 diabetes inUK, ages 25–65, recruitedbetween 1977 and 1991.	Conventional/Intensive	11	3,867	12
UKPDS 80 [24]	Long-term follow up of UKPDS	Conventional/Intensive	5–25	3,867	14
WESDR [20]	Observational study of residentsin diabetes in Wisconsin, USdiagnosed with diabetes after theage of 30, recruited between1979 and 1980	Observational	5–30	1,780	14
Rochester [21]	Observational study of residentsin Rochester, US, diagnosed withdiabetes between 1945 and 1969	Observational	1–30	1,470	16
ACCORD [25]	Clinical study of patients with type2 diabetes, ages 40–79 with HbA1cover 7.5% and CVD or ages 55–79with atherosclerosis, albuminuria,left ventricular hypertrophy, or atleast two additional CVD risk factors	Conventional/Intensive	3.5	10,251	8
ADOPT [29]	Clinical study of patients with type2 diabetes from US, Canada orEurope with no pharmaceuticaltreatment, ages 30–75 (Onlypatients treated with metformin orglyburide are included in the validation)	Metformin/Glyburide	4	2,895	10
ADVANCE [26]	Clinical study of patients with type 2diabetes from 20 countries, ages 55or older with a history of majormicrovascular or macrovasculardisease or at least one other riskfactor for vascular disease	Standard/Intensive	5	11,140	10
ASPEN [27]	Clinical study of patients with type2 diabetes, ages 40–75 years (onlyprimary prevention population isincluded in the validation)	Placebo/Atorvastatin	4	1,905	6
CARDS [28]	Clinical study of patients with type2 diabetes from UK or Ireland withone CVD risk factor but no historyof CVD, ages 40–75	Placebo/Atorvastatin	4	2,838	10
Osaka [30]	Observational study of residentswith diabetes in Osaka, Japan,diagnosed between 1960 and 1979,ages 35 or older	Observational	5–20	1,939	32

The table contains the name of the study, a brief description of the patient population, the reported mean or median duration of the study, the treatment groups included in the validation, the number of participants at baseline and the number of endpoints used in the validation.

Data from two of the studies were used in the construction of microvascular complications in the IHE Cohort Model of Type 2 Diabetes, the Wisconsin Epidemiologic Study of Diabetic Retinopathy (WESDR) [20] and a population based study in Rochester, Minnesota (the Rochester Epidemiology Project) [21], and are thus “dependent” validation studies. Two studies based on the observational Swedish NDR [8], [22] qualify as “dependent” validation studies in simulations using the Swedish NDR macrovascular risk equations, but qualify as “independent” validation studies in simulations using either of the UKPDS risk equations. Two studies based on the UK Prospective Diabetes Study (UKPDS) data [23], [24] are clearly “dependent” in simulations using the UKPDS risk equations. They are also “dependent” in simulations using the Swedish NDR macrovascular risk equations, however, as the UKPDS mortality equations are used (there are currently no complete mortality equations with Swedish NDR data). The ISPOR/SMDM recommendations do note that data sources can have different degrees of “dependence” for different outcomes, though, with a greater degree of dependence for those outcomes actually directly estimated using the risk equations and a lesser degree when the relationship is indirect (e.g., UKPDS mortality is clearly inter-related with the risks of events based on other data sources since one must be alive to experience them) [5].

Six of the validation studies are unambiguously “independent”. Four of them are randomized controlled trials that were featured in the 4th and 5th Mt. Hood Challenges [12], [19]: the Action to Control Cardiovascular Risk in Diabetes (ACCORD) [25], the Action in Diabetes and Vascular Disease: Preterax and Diamicron Modified Release Controlled Evaluation (ADVANCE) [26], the Atorvastatin Study for Prevention of Coronary Heart Disease Endpoints in non-insulin-dependent diabetes mellitus (ASPEN) [27], the Collaborative Atorvastatin Diabetes Study (CARDS) [28]. We also selected the A Diabetes Outcome Progression Trial (ADOPT) [29], which is frequently sourced in health economic evaluation for long-term glycemic durability and it was included in the validation of the CDC-RTI model [16]. To expand geographically and to include more long-term mortality data, we included an observational mortality study from Japan [30], which has been included in the validation of the CDM [15], the CDC-RTI-model [16] and the ECHO-T2DM model [18].

For two of the studies, outcomes were reported separately for patient sub-groups. Specifically, one study based on the Swedish NDR [8] and the study from Japan [30] included four age groups for women and four for men respectively We treated each of the sub-groups as a separate unit of analysis, thus increasing the number of outcomes per study. In addition, one study from UKPDS and the Japanese study included survival at different points in time, each of which was included as a separate unit of analysis as well.

### Endpoints

All endpoints in each study for which a corresponding outcome exists in the model were included in the validation. Composite endpoints, such as “major microvascular events” in ADVANCE [26] were excluded since no match existed in the model. Further, only endpoints reported as a cumulative incidence were included. For example, macroalbuminuria in the UKPDS study was excluded since it was reported as prevalence among non-censored patients [23]. In total, we simulated the cumulative incidence for 167 microvascular, macrovascular, and mortality endpoints). The number of endpoints contributed by each of the studies is included in Table 1. The full list of endpoints included in this analysis is included in the Supporting Information: Table S2.

### Statistical Analysis

We followed established validation methods (for example, see CDM [15], CDC-RTI model [16] and ECHO-T2DM [18]). First, the predicted cumulative incidences were plotted against the observed cumulative incidences for visual inspection. When the predictions match the observed values exactly (i.e., perfect concordance), the validation points will fall along the identity (45°) line. General overprediction is reflected in a preponderance of points above the identity line and underprediction in points below the identity line. Secondly, to quantify the results, we used linear regression analysis (with heteroskedasticity-consistent standard errors based on the Huber-White estimator to account for possible serial dependence when multiple outcomes were taken from the same data source) to estimate the intercept and slope coefficients of the best-fitting line as well as the coefficient of determination (*R* ^2^). Specifically, we fit the following equation:
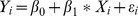
where *Y*_i_ is the predicted cumulative incidence for endpoint i, *X*_i_ the observed cumulative incidence for endpoint i, β_0_ the intercept, β_1_ the slope and ε_i_ the disturbance term. A perfect match is characterized by an intercept of 0, a slope coefficient of 1, and a perfect *R* ^2^ (i.e., 1.00). Note, as the estimated regression coefficients reflect the best-fitting line through the scatterplot but not the points specifically, it is possible that none of the actual points falls close to the identity line (some too high, others too low) but that the regression line coincides with the identity line anyway, dictating that the *R* ^2^ (i.e., how close the sample points are to the regression line) must be considered as well.

Our main analysis includes the full set of 167 validation endpoints. We also assessed concordance separately for the “dependent” and “independent”, where “independent” endpoints are naturally more challenging. The validation methodology is a natural way to assess the performance of individual parts of the model, so we have also assessed concordance separately by type of outcome (mortality, microvascular endpoints, and macrovascular endpoints).

## Results

**Figure 1:**
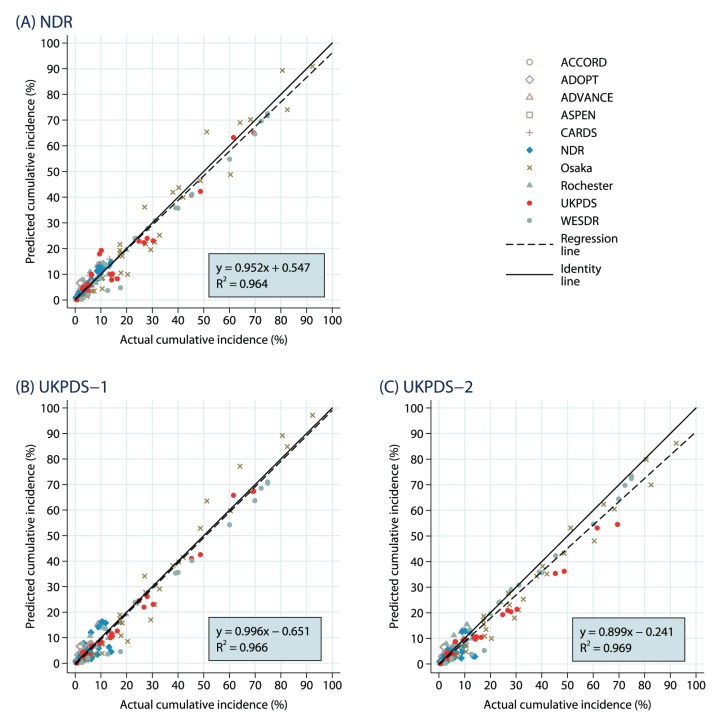
All Endpoints: Predicted Vs. Observed Cumulative Incidence. The results are shown separately for (A) NDR equations, (B) UKPDS-1 equations and (C) UKPDS-2 equations.

Results for the main analysis including all 167 outcomes are summarized in scatterplots in Figures 1(A) to 1(C) for simulations using macrovascular risk equations using the Swedish NDR, UKPDS-OM1, and UKPDS-OM2, respectively. Numerical results can be found in Supporting Information: Table S2. Predicted cumulative incidences are plotted on the vertical axis and the values actually observed in the trial are plotted on the horizontal axis. The data points for each trial are depicted with different symbol and color combinations. For each of the three sets of simulation results, the points visually follow the identity (*solid black*) line, with some points above (overestimates) and some points below (underestimates) but most quite close. There is a preponderance of data points located at relatively small cumulative incidences (for example, 20% or below), reflecting the relatively short time horizon in some of the trials and the rarity of some of the outcomes, though there are also data points throughout the distribution including large values from the UKPDS, WESDR, Rochester, and Osaka studies.

The best-fitting regression lines through the scatterplots each have high *R* ^2^ values (between 0.964 and 0.969), reflecting the linearity of the sample points (i.e., the points lie generally close to the regression line). Though all are reasonably near the values of 0 for the intercept and 1 for the slope, the estimated regression coefficients do vary between the three sets of simulations. The analysis using the UKPDS-OM1 risk equations follows the identity line almost coincidentally, with a slope of 0.996. Using the UKPDS-OM2 risk equations, however, produced a general tendency to underestimate the outcomes (slope of 0.899), interestingly including most of the endpoints from the UKPDS study itself (*the red circles*). The analysis using the Swedish NDR risk equations had a slight tendency to underestimate outcomes (slope of 0.952), but the best-fitting regression line closely mirrored the identity line and no clear pattern is noticeable.

### ‘Dependent’ vs. ‘Independent’ Outcomes

Dividing the outcomes into dependent and independent with respect to model construction can shed light both on whether the model is correctly implemented (a model should naturally be able to predict accurately outcomes from studies on which much of the model is based) and on whether the model can predict outcomes accurately across a variety of settings that can be considered “out of sample” (usually a more difficult challenge).

**Figure 2:**
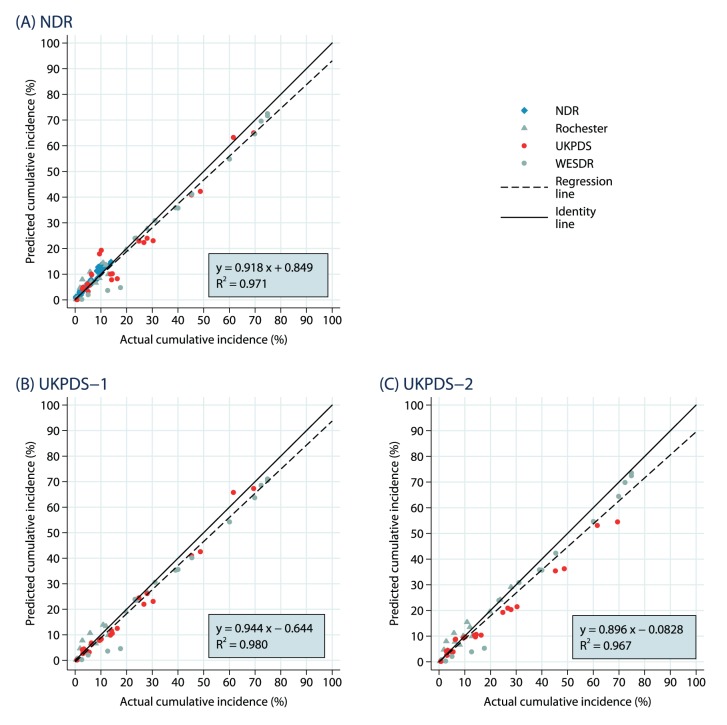
Dependent Endpoints: Predicted Vs. Observed Cumulative Incidence. The results are shown separately for (A) NDR equations, (B) UKPDS-1 equations and (C) UKPDS-2 equations.

**Figure 3:**
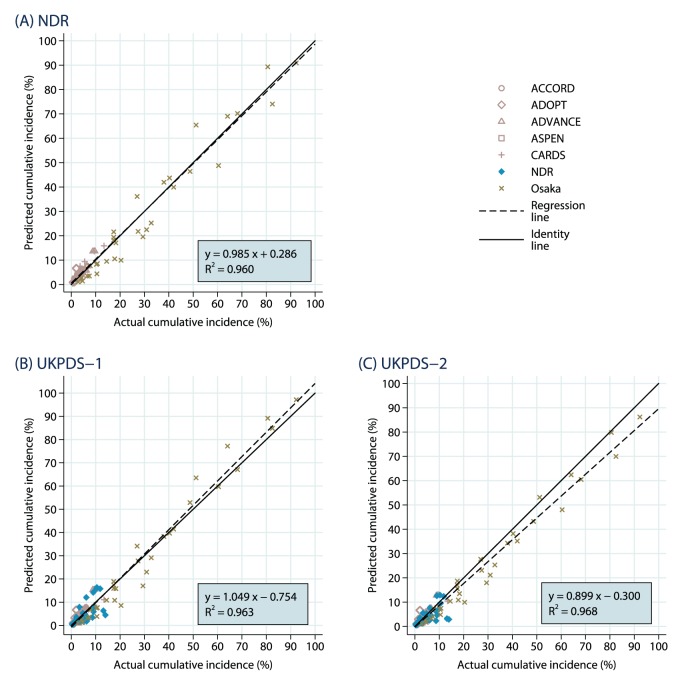
Independent Endpoints: Predicted Vs. Observed Cumulative Incidence. The results are shown separately for (A) NDR equations, (B) UKPDS-1 equations and (C) UKPDS-2 equations.

The results of the subset of dependent outcomes and independent outcomes are presented in Figures 2(A) to 2(C) and Figures 3(A) to 3(C), respectively. Given smaller samples sizes, there is naturally more uncertainty, but the *R* ^2^ values are all at least 0.96 indicating a high degree of linearity.

There are some interesting differences between the dependent and independent analyses for the simulations based on the Swedish NDR and the UKPDS-OM1 macrovascular risk equations. There is a tendency to underpredict the dependent outcomes in the Swedish NDR analyses (slope of 0.918), largely attributable to two WESDR microvascular events and a slight tendency to underpredict outcomes from the UKPDS (included because UKPDS mortality equations were used). The NDR outcomes were all close to the identity line. The subset of independent outcomes were generally closer to the identity line (with a slope of 0.985) for the Swedish NDR simulations, somewhat better than the fit for the dependent outcomes (likely attributable to the classification of the UKPDS outcomes as “dependent”).

The same tendency to underpredict dependent outcomes was observed for the analysis using the UKPDS-OM1 macrovascular risk equations (with a slope of 0.944), driven primarily by the microvascular outcomes from WESDR. There was a slight tendency to overpredict the independent outcomes (with a slope of 1.049), driven largely by the mortality events in the Osaka study. The fit for the Swedish NDR outcomes was spotty, though there was both overprediction and underprediction and no clear pattern.

There was almost no difference between the results for the subsets of dependent and independent outcomes for simulations based on the UKPDS-OM2 macrovascular risk equations, with the same tendency to underprediction in both (slopes of 0.896 and 0.899, respectively). The underprediction in the dependent analyses was largely driven by the UKPDS outcomes, which may be natural as half of the outcomes were drawn from the original UKPDS study (with which the UKPDS-OM1 was estimated). The UKPDS-OM2 was estimated with both the original study data and the more recent UKPDS follow-on study data (and is generally thought to capture recent advances in the treatment of macrovascular disease better).

### Types of Outcomes

Separate analysis of different parts of the model can provide useful insight into the functioning of the model.

#### Mortality

**Figure 4:**
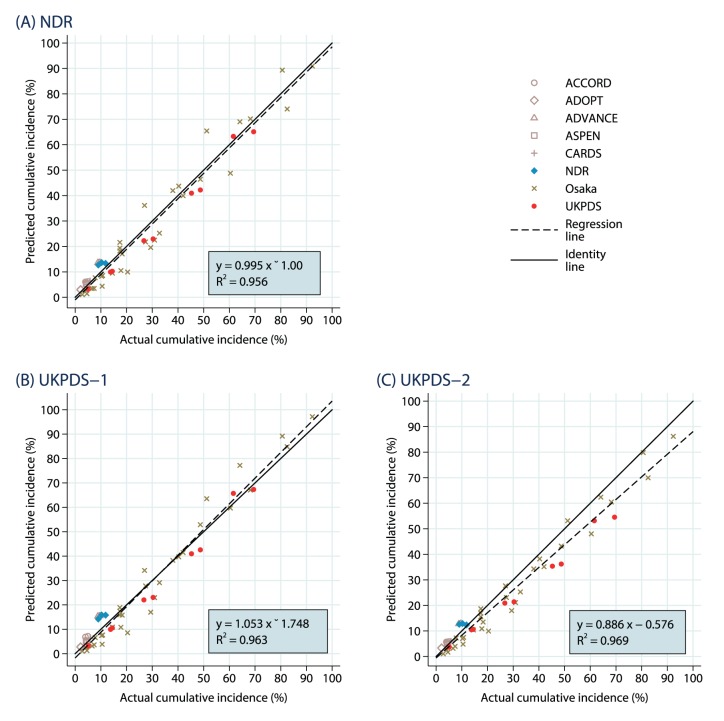
Mortality Endpoints: Predicted Vs. Observed Cumulative Incidence. The results are shown separately for (A) NDR equations, (B) UKPDS-1 equations and (C) UKPDS-2 equations.

The results for the 55 mortality outcomes are summarized as scatterplots in Figures 4(A) to 4(C). The data points are drawn heavily the Osaka study (32) and the UKPDS (10) with relatively long follow-up periods (20 and 25 years, respectively). While there was a tendency for the simulations using the UKPDS-OM2 macrovascular risk equations to underpredict the actual outcomes (slope of 0.886), driven largely by the UKPDS outcomes, the fit was good for analysis with the NDR macrovascular risk equations (slope of 0.995) and for analysis with the UKPDS-OM1 risk equations (slope of 1.053). The R^2^ values were each at least 0.956.

#### Microvascular

**Figure 5:**
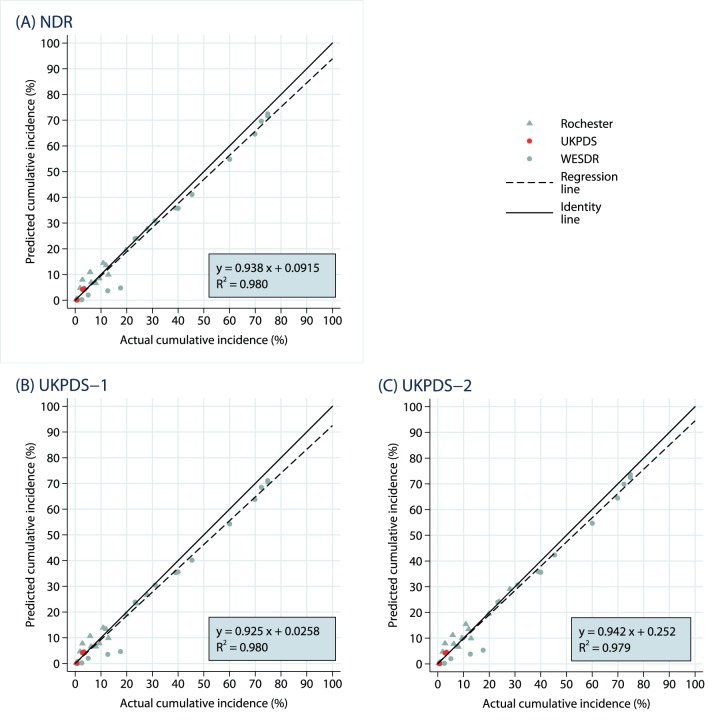
Microvascular Endpoints: Predicted Vs. Observed Cumulative Incidence. The results are shown separately for (A) NDR equations, (B) UKPDS-1 equations and (C) UKPDS-2 equations.

The results for the 34 microvascular outcomes are summarized as scatterplots in Figures 5(A) to 5(C) and are drawn almost entirely from the Rochester and WESDR studies. The differences across analyses using the three sets of macrovascular risk equations are naturally small given that the macrovascular outcomes are excluded. There was a tendency to underpredict the WESDR outcomes and overpredict the Rochester outcomes. The R^2^ values were each about 0.98.

#### Macrovascular

**Figure 6:**
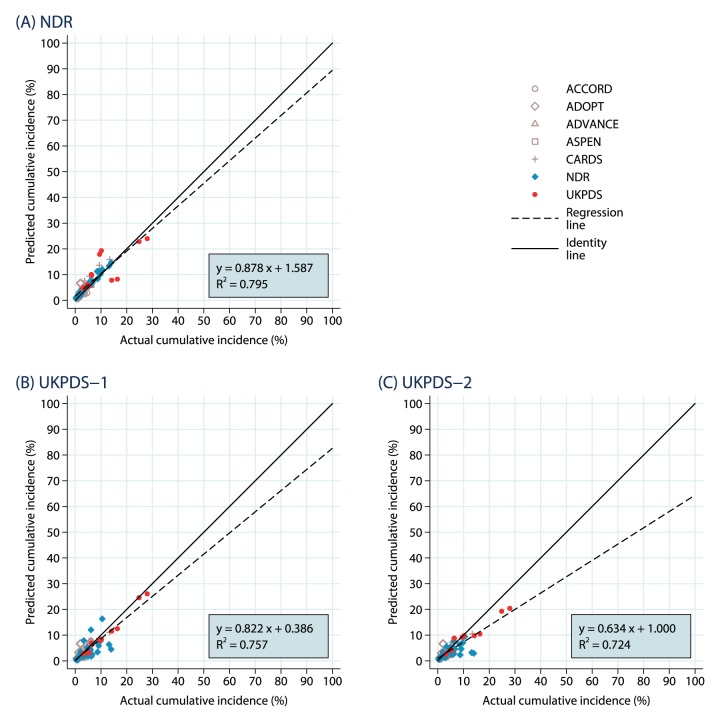
Macrovascular Endpoints: Predicted Vs. Observed Cumulative Incidence. The results are shown separately for (A) NDR equations, (B) UKPDS-1 equations and (C) UKPDS-2 equations.

The results for the 78 macrovascular outcomes are summarized as scatterplots in Figures 6(A) to 6(C). The data points are drawn heavily from the Swedish NDR (32) and the UKPDS (12). The analysis using the Swedish NDR macrovascular risk equations provided the best fit (slope of 0.878), perhaps not surprising given the preponderance of Swedish NDR outcomes, though each of the sets of analyses tended to underpredict actual outcomes. The predictions based on UKPDS-OM1 underestimated most NDR and some of the UKPDS outcomes rendering a slope coefficient of 0.822. The predictions based on the UKPDS-OM2 demonstrated the greatest degree of underestimation, affecting both the NDR and UKPDS outcomes, rendering a slope coefficient of 0.634. The R^2^ values ranged between 0.724 and 0.795, indicating the presence of more outliers than the other outcome sub-groups.

To identify the drivers of this underestimation, we further examined the macrovascular validation points in three categories: Swedish NDR outcomes, UKPDS outcomes, and those from the sub-set of other RCT’s. The results are not reported here but are available on request. Briefly, the simulations using the Swedish NDR risk equations fit the Swedish NDR outcomes (as expected) well, with a slope of 1.015. They also fit the RCT (excluding UKPDS) sub-sample quite well, with a slope of 1.15. There was a considerable degree of underprediction of the UKPDS outcomes, however, which may be natural given the prevalence of better preventive cardiovascular care in the more recent Swedish NDR data. Interestingly, though, both sets of UKPDS risk equations seriously underpredicted the Swedish NDR outcomes (slopes of 0.585 and 0.333, respectively) as well, especially for women and especially for CHF and with a clear relationship to age (fitting better at younger ages). The UKPDS risk engines also underestimated outcomes in the RCT’s, though by less (slopes of 0.852 and 0.769), respectively. The UKPDS-OM1 risk equations fit the UKPDS outcomes best, with a slope of 0.954, while the UKPDS-OM2 risk equations underpredicted with a slope of 0.666.

## Discussion

The IHE Cohort Model of Type 2 Diabetes was subjected to validation testing against 167 endpoints taken from 12 heterogeneous clinical studies, including RCT’s and observational registries; data from the US, Europe, Japan, as well as multi-national RCT’s; and differing chronologically and with length of follow-up. Because the IHE Cohort Model of Type 2 Diabetes supports three different sets of macrovascular risk equations, validity was tested separately for each. The results were positive, with the Swedish NDR and the UKPDS-OM1 validation points closely following the identity line and high *R* ^2^ values. The UKPDS-OM2 validation points had a tendency toward underprediction, driven largely by macrovascular outcomes, but the *R* ^2^ was also high and the best-fitting regression line was relatively close to the identity line.

A look at sub-groups of endpoints found no substantive differences between those that were ‘dependent’ and those that were ‘independent’. Naturally, one expects better prediction for the ‘dependent’ outcomes, though this tendency may be dampened in T2DM by the number of interdependent relationships and use of data from many sources and, for the Swedish NDR risk equations, the classification of the UKPDS endpoints as ‘dependent’ because of the mortality risk equations.

Though the sample sizes were smaller, the predictions for the sub-set of mortality outcomes tracked actual outcomes closely as well, especially the Swedish NDR. The UKPDS-OM2 exhibited a tendency toward underprediction. There is a tendency to underestimate the microvascular outcomes, though this is largely an artifact of two outliers (PDR at 20 and 30 years in WESDR). The similarity across macrovascular risk equations is explained by the exclusion of macrovascular and mortality endpoints, for which choice of equations has a direct effect.

Model predictions performed worse for the sub-set of macrovascular outcomes, however, with underprediction especially with the UKPDS-OM2. There were also important differences across the different sets of macrovascular risk equations, perhaps reflecting differences in the data underlying estimation of the risk equations. The Swedish NDR risk equations, for example, were estimated with relatively recent (2003–2008) data from a naturalistic, relatively unselected, prevalence-based sample of patients with varying disease durations.

In contrast, the UKPDS study was a RCT, subject to strict intervention protocols and limited to newly-diagnosed patients, with recruitment between 1977 and 1991 and study follow-up ending in 1997. The effects of recent treatment patterns (e.g., statin therapy) that have improved macrovascular outcomes are, thus, not captured in the UKPDS-OM1 risk equations, which are entirely estimated with data from the UKPDS RCT. A 10-year non-interventional, post-trial monitoring study of UKPDS survivors ending in 2007 did capture these benefits and these new outcomes were combined with the UKPDS trial data to estimate the UKPDS-OM2 risk equations. These differences are not just reflected in the risk coefficients, they are also reflected in many of the trial outcomes we are trying to match in these exercises. Indeed, the Swedish NDR risk equations closely matched outcomes from the sub-sets of Swedish NDR outcomes and of the RCT outcomes (most of which are relatively recent and more reflective of recent treatment advances), but they underpredicted the UKPDS outcomes (approximately half of which were taken from the trial itself and would not reflect the benefits from these advances). Surprisingly, the UKPDS-OM1 equations underestimated the Swedish NDR and, to a lesser extent, the RCT outcomes, as the UKPDS-OM1 equations have been observed to overpredict macrovascular outcomes in several contemporary studies [31]–[35], though the UKPDS-OM1 reliability predicted the UKPDS Outcomes. The UKPDS-OM2 underestimated each of the sub-groups of outcomes, though the predictions best matched for the RCT outcomes.

It should be noted, however, that macrovascular events pose a greater challenge to match than many of the other types of outcomes for a cohort model. Indeed, even though we did accommodate a small degree of patient heterogeneity by simulating outcomes separately for the four combinations of gender and smoking status and computing the weighted average, the use of cohort mean (rather than individual patient) values of the bio-markers and other risk factors in inherently non-linear risk equations creates a risk for bias [4]. In this respect, the Swedish NDR outcomes were sub-divided into gender/age categories (capturing a greater degree of heterogeneity), which mitigates some of this bias. Not capturing this heterogeneity is the trade-off of using a faster cohort approach rather than a slower micro-simulation approach, but these results illustrate the value of the “middle road”, using the cohort approach but simulating a number of the key sub-groups separately (increasing total run time, but not to the extent of a full micro-simulation run). In the simulations here, we simulated separately by gender and smoking status, though for only one of the validation studies [8] did we have gender-specific baseline characteristics (and for none of them did we have smoking-specific baseline characteristics) so the full effect was not captured. In actual empirical applications, where primary data are at hand and proper sub-group characteristics can be generated, we would suggest that even age categories be included (increasing the number of sub-groups from the current 4 to perhaps 12) given natural non-linearity in macrovascular and particularly mortality patterns.

The results indicate that the choice of macrovascular risk equations can be an important determinant of model results, especially for macrovascular outcomes. Unfortunately, though, it is impossible to know which risk will best fit reality for any given application for which we do not already have results to compare against (as with model validation) and in which case we would presumably have limited use of modeling anyway. This work provides a few clues, however. The Swedish NDR risk equations performed quite well in matching Swedish NDR outcomes (relatively lean Scandinavian patients) and the more recent RCT’s, both of which are characterized by relatively high use of modern preventive medicines such as statins. The UKPDS-OM1 matched best the relatively heavier UK T2DM population without having had the benefit of modern preventive medicine. UKPDS-OM2 uses the same population as UKPDS-OM1 but longer follow-up covering years when modern preventive medicines had become more widely used. Nevertheless, UKPDS-OM2 underpredicted outcomes in the UKPDS population and even the other populations, suggesting it may be suitable for a relatively low risk population. Fortunately, each of the sets of risk equations performed generally well, and there may be value in having multiple sets of risk equations to allow flexibility in tailoring health economic evaluations to setting. More work is needed to explore this further.

The results presented here are generally in line with validation results from previous validation studies of T2DM models [14]–[18]. For example, the *R* ^2^ values of 0.964–0.969 (depending on choice of macrovascular risk equations) indicates a similar linearity of predictions as the Archimedes model (0.99) [14], the CDM (0.89 and 0.90) [15], [17], ECHO-T2DM (0.95) [18] and CDC-RTI (0.99) [16]. The slope coefficient for the analyses using the UKPDS-OM1 model was almost identical to 1 (0.996), despite an underprediction of some macrovascular events. There was a tendency to underprediction with the Swedish NDR macrovascular risk equations (slope of 0.952), but a look at the scatterplot suggests that it performs satisfactorily; especially for more recent studies (underestimating UKPDS outcomes may be quite natural). The slopes are slightly lower than the CDM (1.019), ECHO-T2DM (1.067) and CDC-RTI (1.001). While some analysts have chosen to restrict the intercept to its theoretical value of β_0_ = 0 [14]–[17], exclusion of the intercept is associated with well-known statistical problems in linear regression, including an *R* ^2^ that is not limited to the [0,1] interval. To ensure that any differences versus other validation applications are not spurious, we ran the regressions without the intercept as well. Empirically, the effect was small and the results are available on request.

A strength of the current analysis is the large number of studies (12) and outcomes included (167) and the substantial heterogeneity of the studies, which provides a broad base for assessing the validity of a model in T2DM. Moreover, inclusion of results for 3 different risk equations provides a degree of confidence that, while there are some differences, there is support for use of each of the sets of risk equations.

A weakness of this analysis, as with previous validation studies, was the lack of published data for some model parameters, which may have led to differences between the simulated cohort and the characteristics of the actual patients in the study. This is particularly difficult for some of the RCTs, for example ACCORD and ASPEN, which had complicated, multi-part inclusion and exclusion criteria. As noted, throughout, the cohort modeling approach imposes further limitations on our ability to capture patient heterogeneity, though the relatively favorable results suggests that the trade-off in performance versus a micro-simulation modeling approach do not appear insurmountable. It should also be noted that this study, like other validation studies in T2DM, focused only on the ability of the model to predict mortality, microvascular and macrovascular complications. Validation of utility and costs was not considered owing to a lack of relevant published data to replicate.

## Conclusions

The IHE Cohort model of Type 2 Diabetes was subjected to extensive validation testing and the results were generally in line with the results of other models of T2DM. As many of these models are much slower micro-simulation models, we have shown that the trade-off in accuracy for speed is not necessarily that large. We also found that there were differences by set of macrovascular risk equations used, but that all performed reasonably well in general (though the UKPDS-OM2 substantially underpredicted the included macrovascular outcomes).

## Supporting Information

File S1 **Description of the IHE Cohort Model of Type 2 Diabetes.**
(DOCX)

Table S1 **Validation Data Sources.**
(DOCX)

Table S2 **Detailed Study Validation Results.**
(DOCX)
