# Supplementary material for: Validation of the IHE Cohort Model of Type 2 Diabetes and the Impact of Choice of Macrovascular Risk Equations
Source: PLoS One. 2014 Oct 13;9(10):e110235. doi: 10.1371/journal.pone.0110235 (PMC4195715; doi:10.1371/journal.pone.0110235)
Supplement: File S1 — Description of the IHE Cohort Model of Type 2 Diabetes. (DOCX) [file pone.0110235.s001.docx]

# File S1. Description of the IHE Cohort Model of Type 2 Diabetes

# Introduction

Type 2 Diabetes Mellitus (T2DM) is a chronic and progressive, multi-organ disease characterized by insulin resistance and disturbances of fat metabolism and a resulting hyperglycemia. While the cause is multi-factorial, overweight and obesity are strongly associated with the disease. T2DM is associated with the development of microvascular and macrovascular complications, including stroke, myocardial infarction (MI), end-stage renal disease (ESRD), lower extremity amputation (LEA), and severe visual loss (SVL). These outcomes are serious and costly and T2DM is a major cause of premature mortality.

Though there is currently no cure for T2DM, good disease management can improve long-term patient outcomes. Even with improved lifestyle patterns, however, most patients will require drug therapy to adequately control their blood glucose levels (HbA1c). Blood glucose values tend to increase over time and patients will eventually require additional agents (often double and triple combination oral agents and eventually insulin) to meet target levels. Treatment escalation often comes with the downsides of increased cost and an increased risk of adverse events, including hypoglycemic events and weight gain.

Chronic and progressive diseases pose a challenge to allocating scarce health care resources efficiently. While the benefits (events and associated costs avoided) and costs of new treatments occur over long time horizons, decisions must be taken based on short-run data. Trials of sufficient duration and power to address these economic questions would by definition generate results long after the information was needed (and at great cost, including delayed access to potentially effective treatment options). Due to the estimates of the value-for-money of new treatments being based on short-run clinical evidence uncertainty is inherent in the results.

As with other chronic and progressive diseases, economic modeling (i.e., mathematical equations that synthesize available data such as short-run clinical trial outcomes, risk equations and progression rates, and known physiological relationships into a coherent, internally consistent framework) is a widely used (and accepted) tool for generating credible long-term health economic data in T2DM to assist decision-makers [[1](#_ENREF_1), [2](#_ENREF_2)].

# Model overview

The IHE Cohort Model of Type 2 Diabetes is a cohort model developed to estimate the cost-effectiveness of treatment intervention in T2DM. It uses Markov health states in order to capture important microvascular and macrovascular complications and premature mortality that may result from T2DM. The cycle length is one year and the maximum time horizon is 40 years. The model is highly flexible as most model parameters are defined by the user in the input sheet. It can be run with either deterministic settings or stochastic settings in order to account for second-order uncertainty regarding the value of the underlying parameters.

The model was constructed in Microsoft® Excel 2013 with the aid of the built in Visual Basic for Applications (VBA). To enhance the flexibility of the model the input sheet contains a large number of parameters, which need to be defined by the user in order to run the model. These include baseline characteristics of the cohort, a treatment algorithm, unit costs, quality of life (QoL) weights, choice of risk equations and a number of supporting parameters.

The baseline characteristics of the cohort are demographics (for instance age, gender and ethnicity) biomarkers (such as HbA1c, blood pressure, blood lipids and BMI) and pre-existing complications (for example microalbuminuria or stroke); all of which are risk factors for complications and premature mortality.

The progression of biomarkers over time is driven by a user-defined treatment algorithm. When medications fails to control HbA1c adequately, doses can be altered and new medications added. The algorithm also includes possible medications for blood pressure, blood lipids and overweight.

The micro- and macrovascular health states in the model were selected to include the most important micro- and macrovascular complications related to T2DM. The model uses two parallel Markov chains. The first Markov chain consists of 120 different microvascular health states which are combinations of stages of retinopathy, nephropathy, and neuropathy. The second Markov chain is made of 100 different macrovascular health states which are combinations of stages of ischemic heart disease (IHD), MI, stroke and congestive heart failure (CHF).

Two identical cohorts are created from the user defined baseline characteristics. Each cohort is assigned a separate treatment. Treatment effects are applied to the biomarkers and the evolution of biomarkers is modelled annually until the predefined time horizon is reached. The development and progression of complications and mortality is modelled next to the evolution of biomarkers. Time varying annual transition probabilities govern the progression of the cohorts between different health states. The transition probabilities are calculated from the characteristics of the cohort (diabetes duration, demographics, biomarkers, etc.); mortality risk equations from UKPDS-1 [[3](#_ENREF_3)] or UKPDS-2 [[4](#_ENREF_4)]; macrovascular risk equations from NDR [[5](#_ENREF_5)], UKPDS-1 [[3](#_ENREF_3)] or UKPDS-2 [[4](#_ENREF_4)]; and microvascular risk equations [[6-8](#_ENREF_6)].

Figure 1: Model Schematics

Outcome measures are applied to the cohort in each cycle. Outcomes include survival, life years, quality adjusted life years (QALYs), direct costs and production losses. Both health gains and costs may be discounted in order to address the time preference for money and health. The treatment algorithm is common to the whole cohort and outcomes related to the treatment algorithm are applied to the entire cohort. Outcomes related to micro- and macrovascular complications are applied separately to each proportion of the cohort.

# Generation of a cohort of hypothetical patients

The user needs to define a hypothetical cohort of T2DM patients by assigning a number of baseline characteristics: demographics; biomarkers; history of complications before diagnosis of diabetes; prevalent diabetes related microvascular complications and prevalent diabetes related macrovascular complications.

The baseline demographic variables are baseline age, gender, ethnicity distribution (Caucasian, Black, Hispanic and American Indian), smoker and baseline diabetes duration. The baseline biomarkers are glycated hemoglobin (HbA1c), systolic blood pressure (SBP), diastolic blood pressure (DBP), total cholesterol (TC), low density lipoprotein (LDL), high density lipoprotein (HDL), triglycerides, body mass index (BMI), heart rate (HR), white blood cell count (WBC) and estimated glomerular fibration rate (eGFR).

The baseline complications before diagnosis are measured in per cent and consist of IHD history, MI history, stroke history, CHF history and atrial fibrillation history. The baseline prevalence of stages of micro- and macrovascular complications related to diabetes are all measured in per cent. The baseline cohort is distributed over the 120 possible microvascular health states and 100 possible macrovascular health states by unweighted multiplication.

# Microvascular complications and risk equations

The microvascular complications are divided into three groups: *retinopathy*, *neuropathy* and *nephropathy*. The model contains six stages of retinopathy, five stages of neuropathy and four stages of nephropathy (Table 1). In total there are 120 microvascular health states which are defined as a combinations of the microvascular complications.

Table 1: Stages of microvascular complications

| Retinopathy | Neuropathy | Nephropathy |
| --- | --- | --- |
| None | None | None |
| Background diabetic retinopathy (BDR) | Symptomatic neuropathy | Microalbuminuria |
| Proliferative diabetic retinopathy (PDR) | Peripheral vascular disease (PVD)* | Macroalbuminuria |
| Macular edema (ME) | Lower extremity amputation (LEA) | End stage renal disease (ESRD) |
| ME and PDR | Post LEA |  |
| Severe vision loss (SVL) |  |  |

*For operational reasons PVD has been included among the neuropathy health states even though clinically it is a macrovascular health complication.

The cohort can begin with stages of microvascular complications at baseline and it can develop them during the course of the model. Transition from one health state to another is governed by microvascular risk equations. The model uses separate risk equations for each stage of retinopathy, nephropathy and neuropathy [[6-8](#_ENREF_6)]. While the microvascular complications differ across the cohort the demographic characteristics and biomarkers are the same for the entire cohort.

# Macrovascular complications and risk equations

In the model the macrovascular complications are divided into four groups: *IHD*, *MI*, *stroke* and *CHF*. The model has two stages of IHD, five stages of MI, five stages of stroke and two stages of IHD (Table 2). In total there are 100 macrovascular health states which are defined as a combinations of the stages of macrovascular complications.

Table 2: Stages of macrovascular complications

| IHD | MI | Stroke | CHF |
| --- | --- | --- | --- |
| None | None | None | None |
| IHD | First MI | First stroke | CHF |
|  | Post first MI | Post first stroke |  |
|  | Subsequent MIs | Subsequent strokes |  |
|  | Post subsequent MIs | Post subsequent strokes |  |

The cohort can begin with stages of macrovascular complications at baseline and it can develop them during the course of the model. Transition from one health state to another is governed by macrovascular risk equations. The model uses separate risk equations for IHD, first MI, subsequent MIs, first stroke, subsequent strokes and CHF. The user is free to choose between three sets of macrovascular risk equations, either NDR [[5](#_ENREF_5)], UKPDS-1 [[3](#_ENREF_3)] or UKPDS-2 [[4](#_ENREF_4)]. While the macrovascular complications differ across the cohort the demographic characteristics and biomarkers are the same for the entire cohort.

# Mortality risk equations

The user can choose between two sets of mortality risk equations, either UKPDS-1 [[3](#_ENREF_3)] or UKPDS-2 [[4](#_ENREF_4)]. The UKPDS-1 uses three separate mortality risk equations to calculate survival: *event mortality***,** *diabetes mortality* and *other mortality*. UKPDS-2 mortality risk equations The UKPDS-2 uses four separate mortality risk equations to calculate survival: *death in years with no history or event*, *death in first year of event*, *death in years with history but no events* and *death in subsequent years of events.*

# Treatment algorithm

A valid treatment algorithm must be assigned to each of the treatment arms. The key feature of the treatment algorithm is to define a sequence of treatments which will be initiated successively to maintain HbA1c below a user-defined threshold level. The treatment steps in the sequence are defined by an HbA1c switch threshold (when the next treatment step is initiated), initial absolute treatment effects on all biomarkers levels (at the start of each treatment step) and annual drift of the biomarker levels (while the treatment is continued). The HbA1c treatment steps also have annual event rates for up to three user-specified grades of hypoglycemia and five other user-specified adverse events.

In addition, there are separate simpler treatment algorithms for management of blood pressure, blood lipids and obesity. Each of these are defined by a biomarker switch threshold (when the treatment is initiated), initial absolute treatment effects on relevant biomarker levels (at the start of the treatment) and relative treatment effects on the relevant biomarker levels (while the treatment is continued).

# Quality of life

The QoL function in the model is inspired by the modelling of quality of life values that was conducted using data from the CODE-2 study [[9](#_ENREF_9)]. They used multivariate regression techniques and a data set of 4,461 T2DM patients to attribute QoL decrements to specific individual patient characteristics, treatment, and health complications. The original equation allowed for separate decrements for a number of microvascular and macrovascular complications. There were also decrements for age (per ten years), diabetes duration (per 10 years), overweight (per BMI over 25) and being female. The original equation also had decrements for tablet and insulin treatment.

In addition, the model equation includes decrements for the micro- and macrovascular complications not present in the original equation. It also includes separate decrements for each HbA1c treatment step, in addition to the decrements for tablet and insulin treatment. Decrements for foot ulcers, depression and the combination of symptomatic neuropathy are included in the original equation but not in the model equation. The baseline QoL and the size of decrements are user-defined. Finally, the model subtracts direct user-specified QALY decrements for each episode of the three user-specified grades of hypoglycemia as well as for each episode of the five user-specified adverse events.

# Costs

For each stage of the microvascular and macrovascular complication there is a parameter for the first year direct cost. If the duration of a stage is longer than one year there is another parameter for the direct cost applied during each subsequent year. Each treatment (HbA1c, blood pressure, blood lipids and obesity) also has a parameter for the yearly direct cost. In addition, there are parameters for episode based direct costs for mild, moderate and severe hypoglycemia as well as each of the five user-specified adverse events.

In order to calculate indirect costs, each complication also has a parameter for a reduction in the proportion of people working, measured in per cent. There are also parameters for episode based indirect costs for mild, moderate and severe hypoglycemia as well as each of the five adverse events. The model also allows for the possibility of including net consumption to expand the scope of the societal perspective.

# Model outcomes

The model generates a number of different outcomes. Life years, QALYs and total costs are reported for both treatment strategies and for the increment between the treatments strategies (both with and without discounting). In addition to the total costs, the costs are reported separately for HbA1c treatment, blood pressure treatment, blood lipid treatment, obesity treatment, hypoglycemia, other adverse events, retinopathy, neuropathy and nephropathy, IHD, MI, stroke, CHF, production loss and net consumption.

The incremental cost effectiveness ratio (ICER) for life years and QALYs are calculated by comparing the incremental cost to incremental gain. The net monetary benefit (NMB) is calculated by multiplying the incremental QALYs by the willingness to pay (WTP) per QALY and subtracting the incremental cost.

The biomarker levels in each cycle for both treatment arms are shown in separate diagrams for all biomarkers. The cumulative incidence rates of micro- and macrovascular complications for each treatment strategy are shown in a table and as separate diagrams for each set of complications (together with the cumulative survival). While the diagrams show the cumulative incidence for all cycles the table only displays one cycle at a time. Event rates of hypoglycemia and other adverse events in each cycle for both treatment arms are illustrated in separate diagrams for the resulting adverse events

# Probabilistic sensitivity analysis

The model uses second order probabilistic sensitivity analysis (PSA) to account for uncertainty in several of the parameters. Note that the second order PSA does not vary the baseline characteristics of the cohort: demographics, biomarkers, history of complications before diagnosis of diabetes, prevalent diabetes related microvascular complications or prevalent diabetes related macrovascular complications.

The absolute treatment effect on the HbA1c level, the annual absolute drift of the HbA1c level and the HbA1c are varied using a normal distribution. The initial absolute treatment effects on other biomarker levels and absolute drift of the other biomarker levels are varied using a normal distribution as well. The event rates for hypoglycemia and other adverse events are varied using a log-normal distribution, in order to avoid negative values.

The UKPDS-1 macro coefficients, NDR macro coefficients and UKPDS-1mortality coefficients are varied by randomly selecting a prefabricated set of coefficients for each run of the model. These prefabricated sets were estimated from primary data, by each research group, and supplied to us. At this time no PSA may be conducted for the UKPDS-2 macro coefficients, UKPDS-2 mortality coefficients or micro coefficients. Future versions of the model will take this into account.

The QoL decrements and the QALY decrements may be varied using a normal distribution. Note that in extreme cases this allows for some negative values. All unit costs (complications, treatments) may be varied using a normal distribution. Note that in extreme cases this allows for some negative values. The LMR was varied using a beta distribution, in order to limit the distribution between zero and one. Average consumption and production values may be varied using a normal distribution. Note that in extreme cases this allows for some negative values.

In the PSA analysis, survival, life years, QALYs, costs, ICERs and NMB are reported as the mean with standard errors. In addition, a cost-effectiveness plane, showing the distribution of incremental costs and utilities, relative to the comparator is also reported. Finally, a cost-effectiveness acceptability curve, illustrating the probability that the intervention arm is a cost-effective alternative to the comparator arm, versus different values for the willingness-to-pay for a QALY is also reported.

# References

1. American Diabetes Association Consensus Panel, *Guidelines for Computer Modeling of Diabetes and its Complications.* Diabetes care, 2004. **27**(9): p. 2262-5.

2. Caro, J.J., et al., *Modeling Good Research Practices-Overview: A Report of the ISPOR-SMDM Modeling Good Research Practices Task Force-1.* Value in health : the journal of the International Society for Pharmacoeconomics and Outcomes Research, 2012. **15**(6): p. 796-803.

3. Clarke, P.M., et al., A model to Estimate the Lifetime Health Outcomes of Patients with Type 2 Diabetes: the United Kingdom Prospective Diabetes Study (UKPDS) Outcomes Model (UKPDS no. 68). Diabetologia, 2004. **47**(10): p. 1747-59.

4. Hayes, A.J., et al., UKPDS outcomes model 2: a new version of a model to simulate lifetime health outcomes of patients with type 2 diabetes mellitus using data from the 30 year United Kingdom Prospective Diabetes Study: UKPDS 82. Diabetologia, 2013. **56**(9): p. 1925-33.

5. Ahmad Kiadaliri, A., et al., Towards renewed health economic simulation of type 2 diabetes: risk equations for first and second cardiovascular events from Swedish register data. PLoS One, 2013. **8**(5): p. e62650.

6. Bagust, A., et al., An Economic Model of the Long-Term Health Care Burden of Type II Diabetes. Diabetologia, 2001. **44**(12): p. 2140-55.

7. Brown, J.B., et al., *The Global Diabetes Model: User Friendly Version 3.0.* Diabetes Research and Clinical Practice, 2000. **50 Suppl 3**: p. S15-46.

8. Eastman, R.C., et al., Model of Complications of NIDDM. I. Model Construction and Assumptions. Diabetes Care, 1997. **20**(5): p. 725-34.

9. Bagust, A. and S. Beale, Modelling EuroQol Health-Related Utility Values for Diabetic Complications from CODE-2 Data. Health Economics, 2005. **14**(3): p. 217-30.
