# Supplementary material for: Validation of the IHE Cohort Model of Type 2 Diabetes and the Impact of Choice of Macrovascular Risk Equations
Source: PLoS One. 2014 Oct 13;9(10):e110235. doi: 10.1371/journal.pone.0110235 (PMC4195715; doi:10.1371/journal.pone.0110235)
Supplement: Table S1 — Validation Data Sources. (DOCX) [file pone.0110235.s002.docx]

**Table S1. Data sources used in the external validation of the IHE Cohort Model of Type 2 Diabetes.**

The table contains the references to the studies that were used to load the model. In the cases where no information was found we have made an Assumption, which are marked with an A in the table.

|  | UKPDS | ADVANCE | NDR I | NDR II | ADOPT | CARDS | ACCORD | ASPEN | OSAKA | WESDR | Rochester |
| --- | --- | --- | --- | --- | --- | --- | --- | --- | --- | --- | --- |
| Simulation Duration (Years) | [1] | [2] | [3] | [4] | [5] | [6] | [7] | [8] | A | A | A |
| Baseline Age (Years) | [1] | [2] | [3] | [4] | [5] | [6] | [7] | [8] | A | [9] | [9] |
| Male (%) | [1] | [2] | [3] | [4] | [5] | [6] | [7] | [8] | [10] | [9] | [9] |
| Ethnicity (%) | [1] | A^[[1]](#footnote-1)^ | [3] | A | [5] | A | A | A | A | A | A |
| Caucasian | [1] | A | [3] | A | [5] | A | A | A | A | A | A |
| Black | [1] | A | [3] | A | [5] | A | A | A | A | A | A |
| Hispanic | [1] | A | [3] | A | [5] | A | A | A | A | A | A |
| American Indian | [1] | A | [3] | A | [5] | A | A | A | A | A | A |
| Baseline Duration of Diabetes (Years) | [1] | [2] | [3] | [4] | [5] | [6] | [7] | [8] | [10] | A | A |
| Smoker [0 = No, 1 = Yes] | [1] | [2] | [3] | [4] | [11] | [6] | [7] | [8] | [12] | [1] | [1] |
| Starting Bio-Marker Values |  |  |  |  |  |  |  |  |  |  |  |
| Glycated Haemoglobin (HbA1c, %) | [1] | [2] | [3] | [4] | [5] | [6] | [7] | [8] | [1] | [9] | [13] |
| Systolic Blood Pressure (SBP, mmHg) | [1] | [2] | [3] | [4] | [5] | [6] | [7] | [8] | [10] | [9] | [9] |
| Diastolic Blood Pressure (DBP, mmHg) | [1] | [2] | [3] | [14] | [5] | [6] | [7] | [8] | [10] | [9] | [9] |
| Total Cholesterol (TC, mmol/L) | [1] | [15] | [3] | [16] | [5] | [6] | [7] | [8] | [10] | [1] | [1] |
| Low Density Lipoprotein (LDL, mmol/L) | [1] | [2] | [3] | [16] | [5] | [6] | [7] | [8] | [1] | [1] | [1] |
| High Density Lipoprotein (HDL, mmol/L) | [1] | [2] | [3] | [16] | [5] | [6] | [7] | [8] | [1] | [1] | [1] |
| Triglycerides (mmol/L) | [1] | [2] | [3] | [16] | [5] | [6] | [17] | [8] | [10] | [1] | [1] |
| Body Mass Index (BMI, kg/m^2^) | [1] | [2] | [3] | [4] | [5] | [6] | [7] | [8] | [18] | [9] | [9] |
| Heart rate (HR, bpm) | [19] | [19] | [19] | [19] | [19] | [19] | [19] | [19] | [19] | [19] | [19] |
| White blood cell count (WBC) | [19] | [19] | [19] | [19] | [19] | [19] | [19] | [19] | [19] | [19] | [19] |
| eGFR | [19] | [19] | [19] | [19] | [19] | [19] | [19] | [19] | [19] | [19] | [19] |
| History of Complications Before Diagnoses (%) |  |  |  |  |  |  |  |  |  |  |  |
| Ischaemic Heart Disease (IHD) | [3] | [3] | [3] | [3] | [3] | [3] | [3] | [3] | [3] | [3] | [3] |
| Myocardial Infarction (MI) | [3] | [3] | [3] | [3] | [3] | [3] | [3] | [3] | [3] | [3] | [3] |
| Stroke | [3] | [3] | [3] | [3] | [3] | [3] | [3] | [3] | [3] | [3] | [3] |
| Congestive Heart Failure (CHF) | [3] | [3] | [3] | [3] | [3] | [3] | [3] | [3] | [3] | [3] | [3] |
| Atrial Fibrillation |  |  |  |  |  |  |  |  |  |  |  |
| Age at First Diabetes Related Event (Years) | [1] | [2] | [3] | [4] | [5] | [6] | [7] | [8] | A | [9] | [9] |
| Microvascular complications at baseline |  |  |  |  |  |  |  |  |  |  |  |
| Background Diabetic Retinopathy (BDR) | [1] | [20] | [20] | [20] | [20] | [20] | [20] | [20] | [20] | [21] | [21] |
| Proliferative Diabetic Retinopathy (PDR) | [20] | [20] | [20] | [20] | [20] | [20] | [20] | [20] | [20] | [21] | [21] |
| Macular Oedema (ME) | [20] | [20] | [3] | [20] | [20] | [20] | [20] | [20] | [20] | [21] | [21] |
| Symptomatic neuropathy | [21] | [21] | [21] | [21] | [21] | [21] | [21] | [21] | [21] | [21] | [21] |
| Peripheral Vascular Disease (PVD) | [22] | [22] | [22] | [22] | [22] | [22] | [22] | [8] | [22] | [22] | [21] |
| Microalbuminuria | [1] | [2] | [3] | [3] | [23] | [6] | [6] | [6] | [24] | [21] | [21] |
| Macroalbuminuria | [1] | [2] | [3] | [3] | [23] | [6] | [6] | [6] | [24] | [21] | [21] |
| Macrovascular complications at baseline |  |  |  |  |  |  |  |  |  |  |  |
| Ischaemic Heart Disease (IHD) | [19] | [2] | [3] | [3] | [5] | [6] | [7] | [8] | [3] | [25] | [25] |
| Myocardial Infarction (MI) | [1] | [2] | [3] | [3] | [5] | [6] | [7] | [8] | [3] | [25] | [25] |
| Stroke | [1] | [2] | [3] | [4] | [5] | [6] | [7] | [8] | [3] | [25] | [25] |
| Congestive Heart Failure (CHF) | [19] | [2] | [3] | [4] | [5] | [6] | [7] | A | [3] | [7] | [7] |

# **References**

1. UK Prospective Diabetes Study (UKPDS) Group, *Intensive blood-glucose control with sulphonylureas or insulin compared with conventional treatment and risk of complications in patients with type 2 diabetes (UKPDS 33).* Lancet, 1998. **352**(9131): p. 837-53.

2. Advance Collaborative Group, et al., *Intensive blood glucose control and vascular outcomes in patients with type 2 diabetes.* N Engl J Med, 2008. **358**(24): p. 2560-72.

3. Ahmad Kiadaliri, A., et al., *Towards renewed health economic simulation of type 2 diabetes: risk equations for first and second cardiovascular events from Swedish register data.* PLoS One, 2013. **8**(5): p. e62650.

4. Eeg-Olofsson, K.C., J.; Nilsson, P.M.; Zethelius, B.; Svensson, A.-M.; Gudbjörnsdóttir, S.; Eliasson, B., *New aspects of HbA1c as a risk factor for cardiovascular diseases in type 2 diabetes: an observational study from the Swedish National Diabetes Register (NDR).* Journal of Internal Medicine, 2010. **268**(5): p. 471-482.

5. Kahn, S.E., et al., *Glycemic durability of rosiglitazone, metformin, or glyburide monotherapy.* The New England journal of medicine, 2006. **355**(23): p. 2427-43.

6. Colhoun, H.M., et al., *Primary prevention of cardiovascular disease with atorvastatin in type 2 diabetes in the Collaborative Atorvastatin Diabetes Study (CARDS): multicentre randomised placebo-controlled trial.* Lancet, 2004. **364**(9435): p. 685-96.

7. Gerstein, H.C., et al., *Effects of intensive glucose lowering in type 2 diabetes.* The New England journal of medicine, 2008. **358**(24): p. 2545-59.

8. Knopp, R.H., et al., *Efficacy and safety of atorvastatin in the prevention of cardiovascular end points in subjects with type 2 diabetes: the Atorvastatin Study for Prevention of Coronary Heart Disease Endpoints in non-insulin-dependent diabetes mellitus (ASPEN).* Diabetes care, 2006. **29**(7): p. 1478-85.

9. Klein, R., et al., *The Wisconsin epidemiologic study of diabetic retinopathy. IV. Diabetic macular edema.* Ophthalmology, 1984. **91**(12): p. 1464-74.

10. Sasaki, A.U., M.; Horiuchi, N.; Hasegawa, K.; Shimizu, T., *A E-year follow-up study of patients with non-insulin-dependent diabetes mellitus (NIDDM) in Osaka, Japan.* Diabetes research and clinical practice, 1997. **36**(1): p. 41-47.

11. Viberti, G., et al., *A Diabetes Outcome Progression Trial (ADOPT): baseline characteristics of Type 2 diabetic patients in North America and Europe.* Diabetic medicine : a journal of the British Diabetic Association, 2006. **23**(12): p. 1289-94.

12. Sasaki, A., et al., *Mortality from coronary heart disease and cerebrovascular disease and associated risk factors in diabetic patients in Osaka District, Japan.* Diabetes research and clinical practice, 1995. **27**(1): p. 77-83.

13. Klein, R. and B.E. Klein, *Relation of glycemic control to diabetic complications and health outcomes.* Diabetes care, 1998. **21 Suppl 3**: p. C39-43.

14. Ekstrom, N., et al., *Glucose-lowering treatment and clinical results in 163 121 patients with type 2 diabetes: an observational study from the Swedish national diabetes register.* Diabetes, obesity & metabolism, 2012. **14**(8): p. 717-26.

15. Chalmers, J., *ADVANCE—Action in Diabetes and Vascular Disease: patient recruitment and characteristics of the study population at baseline.* Diabetic medicine : a journal of the British Diabetic Association, 2005. **22**(7): p. 882-888.

16. Eliasson, B., et al., *Clinical usefulness of different lipid measures for prediction of coronary heart disease in type 2 diabetes: a report from the Swedish National Diabetes Register.* Diabetes care, 2011. **34**(9): p. 2095-100.

17. Gerstein, H.C., et al., *Long-term effects of intensive glucose lowering on cardiovascular outcomes.* The New England journal of medicine, 2011. **364**(9): p. 818-28.

18. Yoshiike, N.S., F.; Tajima, S.; Arai, Y.; Kawano, M.; Furuhata, T.; Inoue, S. , *Twenty-year changes in the prevalence of overweight in Japanese adults: The National Nutrition Survey 1976–95.* Obesity Reviews, 2002(3): p. 183-190.

19. Hayes, A.J., et al., *UKPDS outcomes model 2: a new version of a model to simulate lifetime health outcomes of patients with type 2 diabetes mellitus using data from the 30 year United Kingdom Prospective Diabetes Study: UKPDS 82.* Diabetologia, 2013. **56**(9): p. 1925-33.

20. Yau, J.W., et al., *Global prevalence and major risk factors of diabetic retinopathy.* Diabetes care, 2012. **35**(3): p. 556-64.

21. Eastman, R.C., et al., *Model of Complications of NIDDM. I. Model Construction and Assumptions.* Diabetes Care, 1997. **20**(5): p. 725-34.

22. Palumbo, P.J., et al., *Diabetes mellitus: incidence, prevalence, survivorship, and causes of death in Rochester, Minnesota, 1945-1970.* Diabetes, 1976. **25**(7): p. 566-73.

23. Lachin, J.M., et al., *Renal function in type 2 diabetes with rosiglitazone, metformin, and glyburide monotherapy.* Clinical journal of the American Society of Nephrology : CJASN, 2011. **6**(5): p. 1032-40.

24. Sasaki, A., et al., *A 15 year follow-up study of patients with non-insulin dependent diabetes mellitus (NIDDM) in Osaka, Japan. Long-term prognosis and causes of death.* Diabetes research and clinical practice, 1996. **34**(1): p. 47-55.

25. Zhou, H., et al., *A computer simulation model of diabetes progression, quality of life, and cost.* Diabetes care, 2005. **28**(12): p. 2856-63.

1. A = Assumption [↑](#footnote-ref-1)
