# Supplementary material for: Validation of the IHE Cohort Model of Type 2 Diabetes and the Impact of Choice of Macrovascular Risk Equations
Source: PLoS One. 2014 Oct 13;9(10):e110235. doi: 10.1371/journal.pone.0110235 (PMC4195715; doi:10.1371/journal.pone.0110235)
Supplement: Table S2 — Detailed Study Validation Results. (DOCX) [file pone.0110235.s003.docx]

**Table S2. Actual observed cumulative incidence in the studies and predicted cumulative incidence from the model.**

The table contains the name of the studies, the treatment groups (with the age in brackets), the type of endpoint (IHD = ischemic heart disease, MI = myocardial infarction, CHF = congestive heart failure, ESRD = end stage renal disease, BDR = background diabetic retinopathy, PDR = proliferative diabetic retinopathy, LEA = lower-extremity amputation, PVD = peripheral vascular disease), the actual observed cumulative incidence in the studies, the predicted cumulative incidence from the model for all three equations and the difference between observed and predicted cumulative incidence.

| **Study name** | **Treatment group (age)** | **Endpoint** | **Years** | **Observed cumulative incidence** | **Predicted cumulative incidence (%)** | | | **Difference between observed and predicted cumulative incidence (%)** | | |
| --- | --- | --- | --- | --- | --- | --- | --- | --- | --- | --- |
|  |  |  |  |  | **NDR** | **UKPDS-1** | **UKPDS-2** | **NDR** | **UKPDS-1** | **UKPDS-2** |
| NDR (I) | Female (44.7) | IHD | 5 | 1.7 | 2.9 | 1.1 | 1.9 | 1.2 | -0.6 | 0.2 |
| NDR (I) | Female (44.7) | MI | 5 | 1.2 | 1.5 | 1.4 | 1.8 | 0.3 | 0.2 | 0.7 |
| NDR (I) | Female (44.7) | Stroke | 5 | 0.7 | 1.0 | 0.3 | 0.6 | 0.3 | -0.4 | -0.2 |
| NDR (I) | Female (44.7) | CHF | 5 | 0.3 | 0.8 | 0.5 | 0.7 | 0.5 | 0.2 | 0.4 |
| NDR (I) | Female (58.5) | IHD | 5 | 3.1 | 4.3 | 1.5 | 2.4 | 1.2 | -1.6 | -0.7 |
| NDR (I) | Female (58.5) | MI | 5 | 2.1 | 3.7 | 3.1 | 3.5 | 1.6 | 1.0 | 1.4 |
| NDR (I) | Female (58.5) | Stroke | 5 | 1.9 | 3.3 | 1.3 | 1.6 | 1.4 | -0.6 | -0.3 |
| NDR (I) | Female (58.5) | CHF | 5 | 2.4 | 2.6 | 1.8 | 1.4 | 0.3 | -0.6 | -0.9 |
| NDR (I) | Female (69.6) | IHD | 5 | 5.0 | 5.6 | 1.7 | 2.5 | 0.6 | -3.2 | -2.4 |
| NDR (I) | Female (69.6) | MI | 5 | 4.7 | 6.2 | 5.3 | 5.3 | 1.4 | 0.5 | 0.6 |
| NDR (I) | Female (69.6) | Stroke | 5 | 5.0 | 6.3 | 3.2 | 3.1 | 1.3 | -1.8 | -1.9 |
| NDR (I) | Female (69.6) | CHF | 5 | 5.9 | 6.4 | 4.2 | 2.5 | 0.5 | -1.7 | -3.4 |
| NDR (I) | Female (78.7) | IHD | 5 | 6.2 | 6.6 | 1.8 | 2.8 | 0.4 | -4.5 | -3.5 |
| NDR (I) | Female (78.7) | MI | 5 | 9.8 | 10.1 | 7.7 | 7.1 | 0.3 | -2.1 | -2.6 |
| NDR (I) | Female (78.7) | Stroke | 5 | 9.1 | 11.2 | 5.9 | 4.6 | 2.1 | -3.2 | -4.4 |
| NDR (I) | Female (78.7) | CHF | 5 | 13.1 | 13.4 | 6.3 | 3.2 | 0.2 | -6.8 | -9.9 |
| NDR (I) | Male (45.0) | IHD | 5 | 2.1 | 3.7 | 1.9 | 3.4 | 1.6 | -0.1 | 1.3 |
| NDR (I) | Male (45.0) | MI | 5 | 2.1 | 1.9 | 3.6 | 3.2 | -0.2 | 1.5 | 1.1 |
| NDR (I) | Male (45.0) | Stroke | 5 | 0.4 | 1.1 | 0.6 | 0.9 | 0.8 | 0.2 | 0.6 |
| NDR (I) | Male (45.0) | CHF | 5 | 0.7 | 1.1 | 0.5 | 0.6 | 0.4 | -0.2 | -0.1 |
| NDR (I) | Male (58.3) | IHD | 5 | 4.9 | 5.3 | 2.7 | 4.2 | 0.4 | -2.3 | -0.8 |
| NDR (I) | Male (58.3) | MI | 5 | 3.3 | 4.7 | 7.9 | 5.5 | 1.3 | 4.5 | 2.2 |
| NDR (I) | Male (58.3) | Stroke | 5 | 2.6 | 3.5 | 2.1 | 2.4 | 0.9 | -0.5 | -0.2 |
| NDR (I) | Male (58.3) | CHF | 5 | 3.0 | 3.5 | 1.6 | 1.3 | 0.5 | -1.5 | -1.7 |
| NDR (I) | Male (69.4) | IHD | 5 | 6.3 | 6.3 | 2.5 | 4.2 | 0.0 | -3.8 | -2.1 |
| NDR (I) | Male (69.4) | MI | 5 | 6.1 | 7.7 | 12.0 | 7.6 | 1.6 | 5.9 | 1.5 |
| NDR (I) | Male (69.4) | Stroke | 5 | 5.7 | 6.8 | 4.8 | 4.5 | 1.2 | -0.9 | -1.2 |
| NDR (I) | Male (69.4) | CHF | 5 | 8.7 | 8.3 | 3.4 | 2.3 | -0.3 | -5.3 | -6.4 |
| NDR (I) | Male (78.4) | IHD | 5 | 6.6 | 7.4 | 2.5 | 4.6 | 0.8 | -4.1 | -2.0 |
| NDR (I) | Male (78.4) | MI | 5 | 10.5 | 12.0 | 16.3 | 9.5 | 1.5 | 5.8 | -1.0 |
| NDR (I) | Male (78.4) | Stroke | 5 | 8.5 | 11.2 | 7.4 | 6.3 | 2.7 | -1.1 | -2.2 |
| NDR (I) | Male (78.4) | CHF | 5 | 14.0 | 14.7 | 4.5 | 2.9 | 0.7 | -9.5 | -11.1 |
| NDR (II) | HbA1c 6.0-6,9% | Mortality | 6 | 9.0 | 12.7 | 14.2 | 12.6 | 3.7 | 5.2 | 3.6 |
| NDR (II) | HbA1c 7.0-7,9% | Mortality | 6 | 10.3 | 13.7 | 15.9 | 12.9 | 3.4 | 5.6 | 2.6 |
| NDR (II) | HbA1c 8.0-8,9% | Mortality | 6 | 11.8 | 13.3 | 15.8 | 12.4 | 1.5 | 4.0 | 0.5 |
| UKPDS 33 | Conventional | MI | 11 | 16.3 | 8.3 | 12.6 | 10.4 | -8.1 | -3.8 | -5.9 |
| UKPDS 33 | Intensive | MI | 11 | 14.2 | 7.8 | 11.6 | 9.7 | -6.4 | -2.6 | -4.5 |
| UKPDS 33 | Conventional | Stroke | 11 | 4.8 | 6.1 | 3.4 | 4.2 | 1.3 | -1.4 | -0.6 |
| UKPDS 33 | Intensive | Stroke | 11 | 5.4 | 5.6 | 3.2 | 3.9 | 0.2 | -2.3 | -1.5 |
| UKPDS 33 | Conventional | IHD | 11 | 6.3 | 10.1 | 6.8 | 8.8 | 3.7 | 0.5 | 2.4 |
| UKPDS 33 | Intensive | IHD | 11 | 6.5 | 9.8 | 6.3 | 8.8 | 3.3 | -0.2 | 2.3 |
| UKPDS 33 | Conventional | CHF | 11 | 3.2 | 4.9 | 2.7 | 2.5 | 1.7 | -0.4 | -0.7 |
| UKPDS 33 | Intensive | CHF | 11 | 2.9 | 4.6 | 2.6 | 2.6 | 1.7 | -0.3 | -0.3 |
| UKPDS 33 | Conventional | ESRD | 11 | 0.8 | 0.1 | 0.1 | 0.1 | -0.7 | -0.7 | -0.7 |
| UKPDS 33 | Intensive | ESRD | 11 | 0.6 | 0.1 | 0.1 | 0.1 | -0.5 | -0.5 | -0.5 |
| UKPDS 33 | Conventional | Blindness in one eye | 11 | 3.3 | 4.5 | 4.5 | 4.5 | 1.1 | 1.1 | 1.1 |
| UKPDS 33 | Intensive | Blindness in one eye | 11 | 2.9 | 4.2 | 4.2 | 4.2 | 1.3 | 1.3 | 1.3 |
| UKPDS 80 | Conventional | MI | 20 | 28.0 | 24.0 | 26.1 | 20.3 | -4.0 | -1.9 | -7.7 |
| UKPDS 80 | Intensive | MI | 20 | 24.8 | 22.8 | 24.5 | 19.3 | -2.0 | -0.3 | -5.6 |
| UKPDS 80 | Conventional | Stroke | 20 | 10.2 | 19.3 | 8.3 | 9.7 | 9.2 | -1.9 | -0.4 |
| UKPDS 80 | Intensive | Stroke | 20 | 9.5 | 17.9 | 7.8 | 9.2 | 8.4 | -1.7 | -0.3 |
| UKPDS 80 | Conventional | Mortality | 25 | 69.5 | 65.0 | 67.4 | 54.5 | -4.5 | -2.1 | -15.0 |
| UKPDS 80 | Intensive | Mortality | 25 | 61.6 | 63.3 | 65.8 | 53.2 | 1.7 | 4.2 | -8.4 |
| UKPDS 80 | Conventional | Mortality | 20 | 48.7 | 42.2 | 42.5 | 36.3 | -6.5 | -6.2 | -12.4 |
| UKPDS 80 | Intensive | Mortality | 20 | 45.3 | 40.9 | 41.0 | 35.4 | -4.4 | -4.3 | -9.9 |
| UKPDS 80 | Conventional | Mortality | 15 | 30.4 | 23.0 | 23.1 | 21.4 | -7.4 | -7.3 | -9.0 |
| UKPDS 80 | Intensive | Mortality | 15 | 26.7 | 22.3 | 22.0 | 20.9 | -4.4 | -4.7 | -5.8 |
| UKPDS 80 | Conventional | Mortality | 10 | 14.5 | 10.2 | 10.5 | 10.7 | -4.3 | -4.0 | -3.8 |
| UKPDS 80 | Intensive | Mortality | 10 | 13.8 | 10.0 | 9.9 | 10.4 | -3.8 | -3.9 | -3.4 |
| UKPDS 80 | Conventional | Mortality | 5 | 5.1 | 3.2 | 3.4 | 3.8 | -1.9 | -1.7 | -1.3 |
| UKPDS 80 | Intensive | Mortality | 5 | 5.1 | 3.2 | 3.1 | 3.7 | -1.9 | -2.0 | -1.4 |
| WESDR | All | BDR | 5 | 40.0 | 35.7 | 35.6 | 35.6 | -4.3 | -4.4 | -4.4 |
| WESDR | All | BDR | 10 | 60.0 | 54.8 | 54.3 | 54.6 | -5.2 | -5.7 | -5.4 |
| WESDR | All | BDR | 15 | 69.9 | 64.6 | 63.7 | 64.5 | -5.3 | -6.2 | -5.4 |
| WESDR | All | BDR | 20 | 72.3 | 69.5 | 68.5 | 69.8 | -2.8 | -3.8 | -2.5 |
| WESDR | All | BDR | 25 | 74.9 | 71.7 | 70.5 | 72.4 | -3.2 | -4.4 | -2.5 |
| WESDR | All | BDR | 30 | 74.8 | 72.4 | 71.1 | 73.5 | -2.4 | -3.7 | -1.3 |
| WESDR | All | PDR | 5 | 2.5 | 0.2 | 0.2 | 0.2 | -2.3 | -2.3 | -2.3 |
| WESDR | All | PDR | 15 | 5.1 | 2.1 | 2.0 | 2.1 | -3.0 | -3.1 | -3.0 |
| WESDR | All | PDR | 20 | 12.6 | 3.7 | 3.6 | 3.8 | -8.9 | -9.0 | -8.8 |
| WESDR | All | PDR | 30 | 17.6 | 4.8 | 4.6 | 5.3 | -12.8 | -13.0 | -12.3 |
| WESDR | All | Microalbuminuria | 7 | 23.3 | 24.0 | 23.9 | 24.0 | 0.7 | 0.6 | 0.7 |
| WESDR | All | Microalbuminuria | 12 | 31.1 | 30.9 | 30.6 | 30.8 | -0.2 | -0.5 | -0.3 |
| WESDR | All | Microalbuminuria | 17 | 39.0 | 35.8 | 35.3 | 35.9 | -3.2 | -3.7 | -3.1 |
| WESDR | All | Microalbuminuria | 30 | 45.4 | 41.1 | 40.2 | 42.3 | -4.3 | -5.2 | -3.1 |
| Rochester | All | Symptomatic neuropathy | 8 | 12.9 | 10.0 | 9.9 | 9.9 | -2.9 | -3.0 | -3.0 |
| Rochester | All | LEA | 1 | 0.4 | 0.3 | 0.3 | 0.3 | -0.1 | -0.1 | -0.1 |
| Rochester | All | LEA | 2 | 0.8 | 0.7 | 0.7 | 0.7 | -0.1 | -0.1 | -0.1 |
| Rochester | All | LEA | 10 | 1.9 | 4.7 | 4.7 | 4.7 | 2.8 | 2.8 | 2.8 |
| Rochester | All | LEA | 15 | 2.8 | 8.0 | 7.8 | 8.0 | 5.2 | 5.0 | 5.2 |
| Rochester | All | LEA | 20 | 5.8 | 11.0 | 10.7 | 11.2 | 5.2 | 4.9 | 5.4 |
| Rochester | All | LEA | 25 | 10.9 | 14.5 | 14.0 | 15.5 | 3.6 | 3.1 | 4.6 |
| Rochester | All | Macroalbuminuria | 5 | 8.0 | 6.7 | 6.6 | 6.6 | -1.3 | -1.4 | -1.4 |
| Rochester | All | Macroalbuminuria | 10 | 12.0 | 13.6 | 13.4 | 13.5 | 1.6 | 1.4 | 1.5 |
| Rochester | All | Macroalbuminuria | 15 | 19.8 | 19.6 | 19.2 | 19.5 | -0.2 | -0.6 | -0.3 |
| Rochester | All | Macroalbuminuria | 20 | 23.8 | 23.9 | 23.3 | 24.2 | 0.1 | -0.5 | 0.4 |
| Rochester | All | Macroalbuminuria | 30 | 28.0 | 27.7 | 26.7 | 29.1 | -0.3 | -1.3 | 1.1 |
| Rochester | All | ESRD | 10 | 0.5 | 0.2 | 0.2 | 0.2 | -0.3 | -0.3 | -0.3 |
| Rochester | All | ESRD | 20 | 3.3 | 4.2 | 4.1 | 4.4 | 0.9 | 0.8 | 1.1 |
| Rochester | All | ESRD | 25 | 6.2 | 7.0 | 6.6 | 7.8 | 0.8 | 0.4 | 1.6 |
| Rochester | All | ESRD | 30 | 9.4 | 8.5 | 7.8 | 10.1 | -0.9 | -1.6 | 0.7 |
| ACCORD | Standard | MI | 4 | 3.6 | 3.2 | 3.3 | 2.5 | -0.4 | -0.3 | -1.1 |
| ACCORD | Intensive | MI | 4 | 3.6 | 2.8 | 2.8 | 2.3 | -1.8 | -1.8 | -2.3 |
| ACCORD | Standard | Stroke | 4 | 1.2 | 2.5 | 1.2 | 1.2 | 1.2 | -0.1 | -0.1 |
| ACCORD | Intensive | Stroke | 4 | 1.3 | 2.3 | 1.0 | 1.1 | 1.1 | -0.2 | -0.1 |
| ACCORD | Standard | CHF | 4 | 2.4 | 4.2 | 1.5 | 1.3 | 1.2 | -1.5 | -1.7 |
| ACCORD | Intensive | CHF | 4 | 3.0 | 4.2 | 1.3 | 1.3 | 1.8 | -1.1 | -1.1 |
| ACCORD | Standard | Mortality | 4 | 4.0 | 6.2 | 7.2 | 5.8 | 1.2 | 2.2 | 0.8 |
| ACCORD | Intensive | Mortality | 4 | 5.0 | 6.1 | 6.9 | 5.7 | 2.1 | 2.9 | 1.7 |
| ADOPT | Metformin | MI | 4 | 1.6 | 1.4 | 3.4 | 3.0 | -0.2 | 1.9 | 1.4 |
| ADOPT | Glyburide | MI | 4 | 1.2 | 1.4 | 3.3 | 2.9 | 0.1 | 2.0 | 1.6 |
| ADOPT | Metformin | Stroke | 4 | 1.3 | 1.1 | 0.9 | 1.1 | -0.2 | -0.4 | -0.2 |
| ADOPT | Glyburide | Stroke | 4 | 1.2 | 1.1 | 0.9 | 1.1 | -0.1 | -0.3 | -0.1 |
| ADOPT | Metformin | CHF | 4 | 1.3 | 1.0 | 1.1 | 1.1 | -0.3 | -0.2 | -0.2 |
| ADOPT | Glyburide | CHF | 4 | 0.6 | 0.9 | 1.0 | 1.0 | 0.3 | 0.4 | 0.4 |
| ADOPT | Metformin | PVD | 4 | 1.9 | 6.6 | 6.7 | 6.6 | 4.7 | 4.8 | 4.7 |
| ADOPT | Glyburide | PVD | 4 | 2.2 | 6.6 | 6.7 | 6.6 | 4.4 | 4.5 | 4.4 |
| ADOPT | Metformin | Mortality | 4 | 2.1 | 3.0 | 2.8 | 3.3 | 0.9 | 0.7 | 1.2 |
| ADOPT | Glyburide | Mortality | 4 | 2.2 | 3.0 | 2.7 | 3.3 | 0.8 | 0.5 | 1.1 |
| ADVANCE | Conventional | Major coronary event | 5 | 6.1 | 5.9 | 7.7 | 5.4 | -0.2 | 1.6 | -0.7 |
| ADVANCE | Intensive | Major coronary event | 5 | 5.6 | 5.7 | 7.5 | 5.2 | 0.1 | 1.9 | -0.4 |
| ADVANCE | Conventional | Major cerebral event | 5 | 4.4 | 5.3 | 3.0 | 2.8 | 0.9 | -1.4 | -1.6 |
| ADVANCE | Intensive | Major cerebral event | 5 | 4.3 | 5.4 | 2.8 | 2.6 | 1.1 | -1.5 | -1.7 |
| ADVANCE | Conventional | CHF | 5 | 4.1 | 5.1 | 2.5 | 2.0 | 1.0 | -1.6 | -2.1 |
| ADVANCE | Intensive | CHF | 5 | 3.9 | 5.4 | 2.3 | 2.0 | 1.5 | -1.6 | -1.9 |
| ADVANCE | Conventional | PVD | 5 | 6.6 | 7.5 | 7.4 | 7.5 | 0.9 | 0.8 | 0.9 |
| ADVANCE | Intensive | PVD | 5 | 6.2 | 7.5 | 7.4 | 7.6 | 1.3 | 1.2 | 1.4 |
| ADVANCE | Conventional | Mortality | 5 | 9.6 | 13.7 | 15.5 | 12.9 | 4.1 | 5.9 | 3.3 |
| ADVANCE | Intensive | Mortality | 5 | 8.9 | 13.7 | 15.4 | 12.8 | 4.8 | 6.5 | 3.9 |
| ASPEN | Atorvastatin | MI | 4 | 2.9 | 3.7 | 4.5 | 3.6 | 0.8 | 1.6 | 0.7 |
| ASPEN | Placebo | MI | 4 | 3.6 | 3.2 | 3.8 | 3.2 | -0.4 | 0.2 | -0.4 |
| ASPEN | Atorvastatin | Stroke | 4 | 2.8 | 2.6 | 1.4 | 1.8 | -0.2 | -1.4 | -1.0 |
| ASPEN | Placebo | Stroke | 4 | 3.1 | 2.5 | 1.3 | 1.7 | -0.6 | -1.8 | -1.4 |
| ASPEN | Atorvastatin | Mortality | 4 | 4.6 | 5.4 | 5.3 | 5.3 | 0.8 | 0.7 | 0.7 |
| ASPEN | Placebo | Mortality | 4 | 4.3 | 5.3 | 5.1 | 5.1 | 1.0 | 0.8 | 0.8 |
| CARDS | Placebo | MI | 4 | 4.3 | 4.7 | 5.5 | 4.1 | 0.4 | 1.2 | -0.2 |
| CARDS | Atorvastatin | MI | 4 | 2.3 | 3.5 | 4.0 | 3.4 | 1.2 | 1.6 | 1.1 |
| CARDS | Placebo | Stroke | 4 | 2.8 | 3.8 | 2.0 | 2.2 | 1.0 | -0.8 | -0.6 |
| CARDS | Atorvastatin | Stroke | 4 | 1.5 | 3.4 | 1.8 | 1.9 | 1.9 | 0.3 | 0.4 |
| CARDS | Placebo | CHD | 4 | 5.5 | 9.4 | 7.7 | 7.3 | 3.9 | 2.2 | 1.8 |
| CARDS | Atorvastatin | CHD | 4 | 3.6 | 7.6 | 5.5 | 5.8 | 4.0 | 1.9 | 2.2 |
| CARDS | Placebo | CVD | 4 | 13.4 | 15.9 | 11.1 | 10.5 | 2.5 | -2.3 | -2.9 |
| CARDS | Atorvastatin | CVD | 4 | 9.4 | 13.6 | 8.8 | 8.6 | 4.2 | -0.6 | -0.8 |
| CARDS | Placebo | Mortality | 4 | 5.8 | 6.5 | 6.3 | 6.0 | 0.7 | 0.5 | 0.2 |
| CARDS | Atorvastatin | Mortality | 4 | 4.3 | 6.1 | 5.6 | 5.7 | 1.8 | 1.3 | 1.4 |
| Osaka | Male (35-44) | Mortality | 20 | 29.4 | 19.6 | 17.0 | 18.0 | -9.8 | -12.4 | -11.4 |
| Osaka | Male (35-44) | Mortality | 15 | 20.4 | 9.9 | 8.6 | 10.0 | -10.5 | -11.8 | -10.4 |
| Osaka | Male (35-44) | Mortality | 10 | 10.5 | 4.4 | 3.9 | 4.8 | -6.1 | -6.6 | -5.7 |
| Osaka | Male (35-44) | Mortality | 5 | 4.6 | 1.4 | 1.3 | 1.7 | -3.2 | -3.3 | -2.9 |
| Osaka | Male (45-54) | Mortality | 20 | 42.0 | 39.9 | 41.5 | 35.2 | -2.1 | -0.5 | -6.8 |
| Osaka | Male (45-54) | Mortality | 15 | 30.9 | 22.5 | 23.0 | 21.1 | -8.4 | -7.9 | -9.8 |
| Osaka | Male (45-54) | Mortality | 10 | 17.8 | 10.5 | 10.8 | 10.9 | -7.3 | -7.0 | -6.9 |
| Osaka | Male (45-54) | Mortality | 5 | 7.4 | 3.5 | 3.6 | 4.0 | -3.9 | -3.8 | -3.4 |
| Osaka | Male (55-64) | Mortality | 20 | 64.1 | 69.0 | 77.2 | 62.4 | 4.9 | 13.1 | -1.7 |
| Osaka | Male (55-64) | Mortality | 15 | 48.7 | 46.4 | 52.9 | 43.2 | -2.3 | 4.2 | -5.5 |
| Osaka | Male (55-64) | Mortality | 10 | 32.8 | 25.2 | 29.1 | 25.3 | -7.6 | -3.7 | -7.5 |
| Osaka | Male (55-64) | Mortality | 5 | 14.5 | 9.5 | 10.9 | 10.4 | -5.0 | -3.6 | -4.1 |
| Osaka | Male (65+) | Mortality | 20 | 92.3 | 90.9 | 97.2 | 86.2 | -1.4 | 4.9 | -6.1 |
| Osaka | Male (65+) | Mortality | 15 | 82.5 | 74.0 | 84.8 | 70.0 | -8.5 | 2.3 | -12.5 |
| Osaka | Male (65+) | Mortality | 10 | 60.4 | 48.8 | 59.7 | 48.0 | -11.6 | -0.7 | -12.4 |
| Osaka | Male (65+) | Mortality | 5 | 27.4 | 21.8 | 27.8 | 23.1 | -5.6 | 0.4 | -4.3 |
| Osaka | Female (35-44) | Mortality | 20 | 18.3 | 17.1 | 15.8 | 13.5 | -1.2 | -2.5 | -4.8 |
| Osaka | Female (35-44) | Mortality | 15 | 10.3 | 8.3 | 7.5 | 7.0 | -2.0 | -2.8 | -3.3 |
| Osaka | Female (35-44) | Mortality | 10 | 6.6 | 3.5 | 3.2 | 3.2 | -3.1 | -3.4 | -3.4 |
| Osaka | Female (35-44) | Mortality | 5 | 2.8 | 1.1 | 1.0 | 1.1 | -1.7 | -1.8 | -1.7 |
| Osaka | Female (45-54) | Mortality | 20 | 27.0 | 36.1 | 34.1 | 27.7 | 9.1 | 7.1 | 0.7 |
| Osaka | Female (45-54) | Mortality | 15 | 17.4 | 19.4 | 17.4 | 15.5 | 2.0 | 0.0 | -1.9 |
| Osaka | Female (45-54) | Mortality | 10 | 10.7 | 8.6 | 7.7 | 7.5 | -2.1 | -3.0 | -3.2 |
| Osaka | Female (45-54) | Mortality | 5 | 4.2 | 2.7 | 2.5 | 2.6 | -1.5 | -1.7 | -1.6 |
| Osaka | Female (55-64) | Mortality | 20 | 51.2 | 65.4 | 63.5 | 53.1 | 14.2 | 12.3 | 1.9 |
| Osaka | Female (55-64) | Mortality | 15 | 38.0 | 42.0 | 38.3 | 34.3 | 4.0 | 0.3 | -3.7 |
| Osaka | Female (55-64) | Mortality | 10 | 17.4 | 21.6 | 18.9 | 18.7 | 4.2 | 1.5 | 1.3 |
| Osaka | Female (55-64) | Mortality | 5 | 7.8 | 7.7 | 6.6 | 7.3 | -0.1 | -1.2 | -0.5 |
| Osaka | Female (65+) | Mortality | 20 | 80.6 | 89.3 | 89.2 | 79.8 | 8.7 | 8.6 | -0.8 |
| Osaka | Female (65+) | Mortality | 15 | 68.2 | 70.2 | 67.1 | 60.5 | 2.0 | -1.1 | -7.7 |
| Osaka | Female (65+) | Mortality | 10 | 40.3 | 43.7 | 39.7 | 38.2 | 3.4 | -0.6 | -2.1 |
| Osaka | Female (65+) | Mortality | 5 | 17.4 | 18.2 | 15.9 | 16.9 | 0.8 | -1.5 | -0.5 |
